# A zero-inflated beta mixture model for marginal mediation analysis with compositional microbiome mediators

**DOI:** 10.3389/fcimb.2026.1877170

**Published:** 2026-10-29

**Authors:** Seungjun Ahn, Quran Wu, Alicia Yang, Donald Porchia, Weijia Fu, Zhigang Li

**Affiliations:** 1 Department of Population Health Science and Policy, Icahn School of Medicine at Mount Sinai, New York, NY, United States; 2 Department of Biostatistics, University of Florida, Gainesville, FL, United States; 3 Department of Otolaryngology - Head and Neck Surgery, Icahn School of Medicine at Mount Sinai, New York, NY, United States

**Keywords:** causal inference, EM algorithm, mediation analysis, microbiome data, zero-inflated beta mixture model

## Abstract

The role of the microbiome in disease pathogenesis is an emerging field with strong evidence suggesting that dysbiosis is associated with precancerous and cancerous states. Microbiome data present substantial challenges for causal mediation analysis due to sparsity, compositional constraints, and latent heterogeneity. To address these issues, we propose a zero-inflated beta mixture (ZIBM) method for mediation analysis with compositional microbiome mediators. The proposed method accommodates excess zeros through a zero-inflation component and captures heterogeneity in non-zero relative abundances using a beta mixture distribution. Within the potential-outcomes framework, the ZIBM provides estimates of marginal microbiome-mediated causal effects, and model parameters are estimated using an expectation-maximization algorithm. Simulation studies demonstrate that the ZIBM yields more accurate estimation and reliable inference under conditions commonly observed in microbiome data, compared with existing approaches. An application to a real microbiome study further illustrates its practical utility. These results indicate that the proposed method provides a more flexible and robust statistical framework for mediation analysis involving compositional microbiome data.

## 1 Introduction

Advances in high-throughput sequencing technologies, including 16S ribosomal RNA gene sequencing and metagenomic shotgun sequencing, have enabled comprehensive characterization of microbial communities. These technologies have facilitated investigations into microbiome-mediated mechanisms in complex diseases such as Alzheimer’s disease and cancer (Wang et al., 2019; Jin et al., 2019; Tanoue et al., 2019; Kwak et al., 2024; Bautista et al., 2026). Recent research has focused on the potential role of microbiota in cancer development, particularly for head and neck squamous cell carcinoma and colorectal cancer (CRC) (Kwak et al., 2024; Bautista et al., 2026). Oral microbiome dysbiosis had been associated with environmental exposures, oral hygiene, and periodontal disease, as well as cancer (Mousavi et al., 2022; Kwak et al., 2024). Similarly, there is research that shows that gut microbiome dysbiosis associated with CRC is characterized by enrichment of pathobionts and the loss of protective commensal bacterial species (Bautista et al., 2026). Changes in the microbiome may be linked with reprogrammed immune, metabolic, neural, and endocrine pathways. These changes in the relative abundance of bacterial populations may be associated with nutrition, air pollution, endocrine disrupting chemicals, and other environmental exposures (Mousavi et al., 2022). Therefore, there is growing interest in identifying microbial taxa that mediate associations between risk factors and health outcomes.

Mediation analysis provides a statistical framework for quantifying causal pathways involving intermediate variables. It decomposes the total effect of an exposure into direct and indirect components and has been applied across epidemiology, environmental health sciences, and biomedical research. Within the potential-outcomes framework, mediation analysis allows for nonlinear relationships and interaction effects (VanderWeele, 2009; Imai et al., 2010; VanderWeele, 2015). Comprehensive reviews of mediation methodologies and their assumptions are available in the literature (MacKinnon et al., 2007; VanderWeele, 2016; Lange et al., 2017). Despite these advances, extending mediation analysis to microbiome studies remains challenging due to the unique distributional characteristics of microbiome sequencing data.

Microbiome data are inherently compositional, sparse, and high-dimensional, with a substantial proportion of zero observations (Li, 2018). These zeros may arise from true biological absence or from technical artifacts such as undersampling and detection limits. The coexistence of compositional constraints and zero inflation complicates statistical modeling and inference, particularly in mediation settings. Several approaches have been proposed to analyze the microbiome as a high-dimensional mediator (Sohn and Li, 2019; Wang et al., 2020; Zhang et al., 2019). Among them, Sohn and Li (2019) proposed the compositional mediation model (CCMM) for microbiome studies, which accounts for the compositional structure of microbiome mediators. As a general mediation benchmark, we also consider the causal mediation approach of IKT method (Imai et al., 2010), which provides a framework for estimating indirect effects but is not designed specifically for microbiome mediators. However, effectively accommodating zero-inflated compositional mediators remains an open methodological problem. To address these challenges, the MarZIC framework (Wu et al., 2022) was previously developed to model zero-inflated compositional mediators under the potential-outcomes paradigm. MarZIC enables decomposition of mediation effects into components attributable to binary presence-absence changes and continuous abundance variations, thereby providing interpretable insights into microbiome-mediated mechanisms. Due to the compositional structure of microbiome data, the relative abundance (RA) of each taxon is always linearly correlated with other taxa, and thus it is often difficult to define and describe the mediation effect of a taxon. Therefore, MarZIC proposed a definition of the marginal mediation effect by fitting each taxon separately one by one. Nevertheless, the nonzero microbiome RAs can show heterogeneous shapes across samples, including skewness and multiple modes. Such patterns may arise from latent sample subgroups, different microbial community states, or unmeasured host and environmental factors. Therefore, the single beta distribution used in MarZIC for the nonzero abundance component may be too restrictive in some settings. We use a beta mixture model to relax this assumption and to allow the nonzero component to adapt to these empirical distributional patterns. The single beta model is still included as a special case when the mixture has one component.

In this work, we propose a Zero-Inflated Beta Mixture (ZIBM) model for marginal mediation analysis with compositional microbiome mediators. The proposed model extends the MarZIC framework by incorporating a mixture-based representation that captures excess zeros and latent heterogeneity in microbial compositions. Using a zero-inflated beta mixture formulation, the proposed model improves the modeling of sparse and compositional mediators while preserving interpretability within the potential-outcomes framework. This flexibility leads to more accurate estimation and inference of microbiome-mediated causal effects. The remainder of this article is organized as follows. In Section 2, Subsection 2.1 introduces the proposed model and notation. Subsections 2.2 and 2.3 present estimation and inference procedures. In Section 3, Subsection 3.1 evaluates the performance of the proposed method through simulation studies. Subsection 3.2 demonstrates its utility using a real microbiome data application. Section 4 concludes with a discussion.

## 2 Materials and methods

### 2.1 Notation and model specifications

For simplicity, the subject index is omitted from all notations throughout this paper. We will let *Y*, *M*, and *X* denote the continuous outcome variable, the mediator (i.e., a taxon/ASV/OTU), and the exposure variable, respectively. The outcome *Y* is assumed to depend on *M* and *X* through the following marginal regression model (Wu et al., 2022):

(1) $$Y=\beta_{0}+\beta_{1}M+\beta_{2}{𝟙}_{\left(M>0\right)}+\beta_{3}X+\beta_{4}X{𝟙}_{\left(M>0\right)}+\beta_{5}XM+\epsilon ,$$

where 𝟙_(·)_ denotes an indicator function, the random error *ϵ* follows a normal distribution *N*(0*,δ*), and *β*_1_, *β*_2_, *β*_3_, *β*_4_, and *β*_5_ are regression coefficients. The mediator *M* represents the RA of a microbial taxon, an ASV or an OTU.

The mediation framework involves two pathways arising from the association between *M* and *X*. Under a popular choice of Dirichlet distribution for the composition of microbial communities, the marginal distribution of a taxon is Beta distribution. Here, we extend it to a mixture of Beta distributions to accommodate complexities in real-word data. The *M* is modeled using a zero-inflated Beta mixture (ZIBM) distribution, whose two-part density function is given by

(2) $$f\left(m;\theta \right)=\{\begin{array}{ll} \Delta , & m=0\\ \left(1-\Delta \right)(\sum_{k=1}^{K}\psi_{k}\text{Beta}(m|\mu_{k},\phi )+\left(1-\sum_{k=1}^{K}\psi_{k}\right)\text{Beta}(m|\mu_{K+1},\phi )), & m>0, \end{array}$$

where Δ denotes the probability of observing a zero. The function $\text{Beta}(m\;|\;\mu_{k},\phi )=\frac{m^{\mu_{k}\phi -1}{\left(1-m\right)}^{\left(1-\mu_{k}\right)\phi -1}}{B\left(\mu_{k}\phi ,\left(1-\mu_{k}\right)\phi \right)}$ is the Beta density of the *k*th component of the mixture, *ψ_k_* is the weight of the *k*th component, *B*(·,·) is the Beta function, and *µ_k_* and *ϕ* represent the mean and dispersion parameters, respectively. The mixture components are used to capture heterogeneity in the nonzero relative abundance distribution and are not assumed to have a direct one-to-one biological interpretation. For a fixed number of nonzero beta components, AIC and BIC are computed by the method after model fitting. A direct correlation between the number of components and these performance metrics is not necessarily expected. In practice, users can specify a range for candidate numbers of components and our R package can select the optimal number of components based on AIC or BIC. The relationship between the mediator and the independent variable is modeled using the following regression models:

(3) $$\ln(\frac{\mu_{k}}{1-\mu_{k}})=\alpha_{0k}+\alpha_{1k}X,k=1,\ldots ,K+1$$

(4) $$\ln(\frac{\text{Δ}}{1-\text{Δ}})=\gamma_{0}+\gamma_{1}X.$$

(Equations 1–4) jointly define the full mediation model of the proposed ZIBM approach.

#### 2.1.1 Mechanism for observing zeros of the mediator

For microbiome abundance data, observations that cannot be detected are set to be zero. Consequently, the observed abundance data may contain two types of zeros: true zeros representing taxon absence and false zeros arising from measurement (or technical) failure. Let *M* denote the RA of a microbial taxon and let M^*^ represent its observed value. When the observed value is positive (i.e. M^*^ > 0), we assume that *M* = M^*^. However, when M^*^ = 0, it is not clear whether the true abundance is zero or positive but recorded as zero. In this study, we consider the following mechanism for observing zeros in microbial taxon abundance with these zero characteristics:

(5) $$\mathrm{Pr}(M^{*}=0|M,L)={𝟙}_{\left(ML<1\right)},$$

where *L* is the library size (i.e., sequencing depth) and the product *ML* can be interpreted as the sample absolute abundance (SAA) of a taxon in a sample. We refer to this mechanism as “LOD mechanism” since all SAA values below 1, limit of detection (LOD), cannot be detected.

#### 2.1.2 Marginal mediation effect and direct effect

Within the potential-outcomes (PO) framework (VanderWeele, 2016), we define the natural indirect effect (NIE) and natural direct effect (NDE), where the NIE represents the marginal mediation effect (Wu et al., 2022). The total effect of the exposure variable *X* can be expressed as the sum of the NIE and NDE. Let *M_x_* denote the value of *M* when *X* = *x*, and let *Y_xm_* denote the value of *Y* when (*X,M*) = (*x,m*). The average NIE and NDE corresponding to a change in *X* from *x*_1_ to *x*_2_ are defined as follows:


$$\text{NIE}=E\left[Y_{x_{2}M_{x_{2}}}-Y_{x_{2}M_{x_{1}}}\right],$$

$$\text{NDE}=E\left[Y_{x_{2}M_{x_{1}}}-Y_{x_{1}M_{x_{1}}}\right],$$

where $Y_{x_{2}M_{x_{1}}}$ is a counterfactual outcome. We can then further expand NIE into the following equations:


$$\begin{array}{l} \text{NIE}=E[Y_{x_{2}M_{x_{2}}}]-E[Y_{x_{2}M_{x_{1}}}]=E[E(Y_{x_{2}M_{x_{2}}}|M_{x_{2}})]-E[E(Y_{x_{2}M_{x_{1}}}|M_{x_{1}})]\\ =E\left[\beta_{0}+\beta_{1}M_{x_{2}}+\beta_{2}{𝟙}_{\left(M_{x_{2}}>0\right)}+\beta_{3}x_{2}+\beta_{4}x_{2}{𝟙}_{\left(M_{x_{2}}>0\right)}+\beta_{5}x_{2}M_{x_{2}}\right]-E\left[\beta_{0}+\beta_{1}M_{x_{1}}+\beta_{2}1_{\left(M_{x_{1}}>0\right)}+\beta_{3}x_{2}+\beta_{4}x_{2}1_{\left(M_{x_{1}}>0\right)}+\beta_{5}x_{2}M_{x_{1}}\right]\\ =\left(\beta_{1}+\beta_{5}x_{2}\right)\left(E[M_{x_{2}}]-E[M_{x_{1}}]\right)+\left(\beta_{2}+\beta_{4}x_{2}\right)\left(E[{𝟙}_{\left(M_{x_{2}}>0\right)}]-E[{𝟙}_{\left(M_{x_{1}}>0\right)}]\right)\\ ={\text{NIE}}_{1}+{\text{NIE}}_{2}, \end{array}$$

$${\text{NIE}}_{1}=\left(\beta_{1}+\beta_{5}x_{2}\right)\left(E[M_{x_{2}}]-E[M_{x_{1}}]\right),$$

$${\text{NIE}}_{2}=\left(\beta_{2}+\beta_{4}x_{2}\right)\left(\text{expit}\left(\gamma_{0}+\gamma_{1}x_{1}\right)-\text{expit}\left(\gamma_{0}+\gamma_{1}x_{2}\right)\right),$$

where


$$E\left[M_{x_{2}}\right]=\sum_{k=1}^{K}\psi_{k}\left(1-{\text{Δ}}_{x_{2}}\right)\mu_{k_{x_{2}}}+\left(1-\sum_{k=1}^{K}\psi_{k}\right)\left(1-{\text{Δ}}_{x_{2}}\right)\mu_{K+{\text{Δ}}_{x_{2}}},$$

$$E\left[M_{x_{1}}\right]=\sum_{k=1}^{K}\psi_{k}\left(1-{\text{Δ}}_{x_{1}}\right)\mu_{k_{x_{1}}}+\left(1-\sum_{k=1}^{K}\psi_{k}\right)\left(1-{\text{Δ}}_{x_{1}}\right)\mu_{K+{\text{Δ}}_{x_{1}}},$$

$${\text{Δ}}_{x}=\text{expit}\left(\gamma_{0}+\gamma_{1}x\right),$$

$$\mu_{k_{x}}=\text{expit}\left(\alpha_{0k}+\alpha_{1k}x\right),k=1,\ldots ,K+1,$$

where expit(·) is the inverse function of logit(·), *F_Mx_*(*m*) denotes the cumulative distribution function of *M_x_*, and *dF_Mx_*(*m*) denotes the Stieltjes integration (Terhorst, 1986) with respect to *F_Mx_*(*m*). The NIE, NIE_1_, NIE_2_, and NDE can be estimated by substituting the estimated parameters into the corresponding expressions. Confidence intervals (CI) are derived using the multivariate delta method described in the Supplementary Material, while bootstrap procedures provide an alternative means for estimating standard errors and constructing CI (Efron and Tibshirani, 1986). The quantity NIE_1_ represents the mediation effect associated with changes in the mediator on its numeric scale, whereas NIE_2_ captures the mediation effect attributable to the binary status change of the mediator from zero to a non-zero status. This decomposition is presented in **Figure 1** below, which displays two indirect causal pathways from *X* to *Y* through the mediator *M*. Causal mediation analyses requires assumptions to identify counterfactual outcomes (Imai et al., 2010; VanderWeele, 2009). Similar to MarZIC, we make sequential ignorability assumption for our inference.

**Figure 1:**
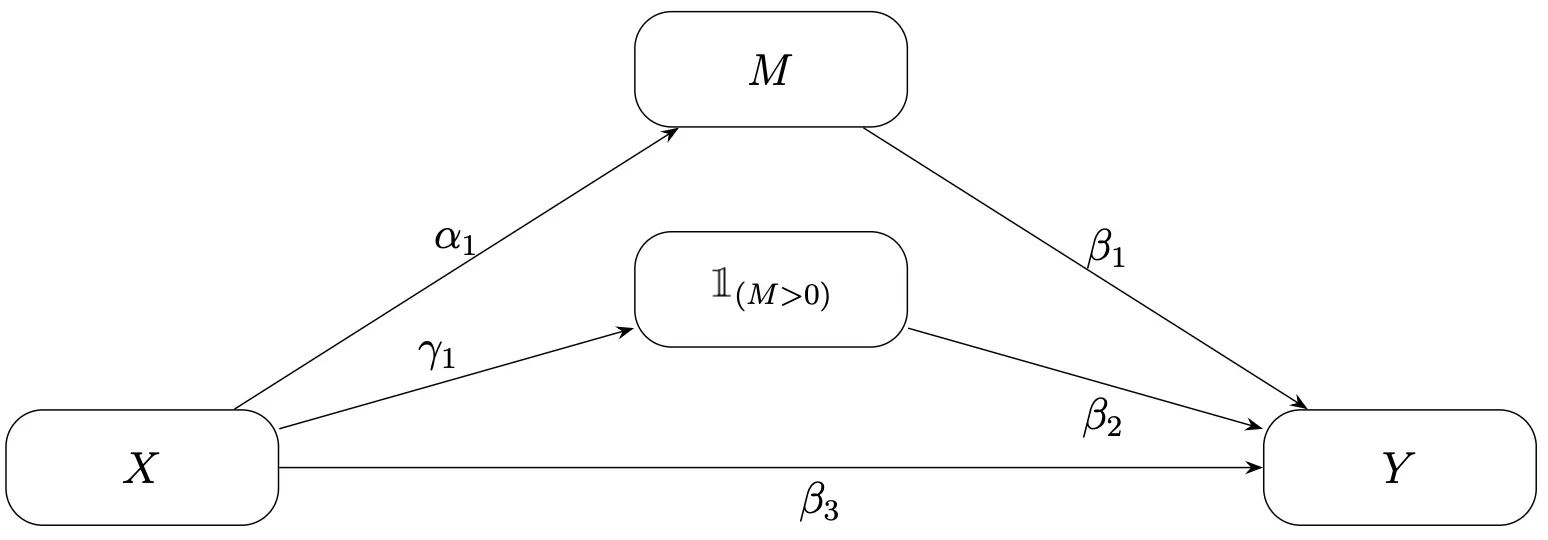
Causal mediation diagram for the ZIBM framework, showing the direct effect of *X* on *Y* and indirect effects through *M* and 𝟙_(*M*>0)_.

### 2.2 Parameter estimation

The indicator variable *c_i_*is defined as follows: *c_i_*= 0, if the true *m* is zero; *c_i_* = *k*, if the true *m* arises from the *k*th Beta distribution. The observed data for each subject can be denoted by the vector (*Y,R,M*^∗^*,L,X*), where *R* = 𝟙_(_*_M_*∗*_>_*_0)_ and the subject index is suppressed. The log-likelihood function

can then be written as follows:


$$\begin{array}{l} \ell =\ln\;(\prod_{i=1}^{N}\prod_{k=0}^{K+1}\left({\text{Ψ}}_{ik}f(y_{i},r_{i},m_{i}^{*}|x_{i},l_{i},c_{i}=k){)}^{{𝟙}_{\left(c_{i}=k\right)}}\right)\\ \;\;\;\;=\sum_{i=1}^{N}\sum_{k=0}^{K+1}{𝟙}_{c_{i}=k}\left(\ln\;({\text{Ψ}}_{ik}\right)+\ln\;(f(y_{i},r_{i},m_{i}^{*}|x_{i},l_{i},c_{i}=k)))\\ \;\;\;\;=\sum_{i=1}^{N}\sum_{k=0}^{K+1}{𝟙}_{c_{i}=k}\left(\ln\;({\text{Ψ}}_{ik}\right)+\ell_{ik}), \end{array}$$

where Ψ*_i_*_0_ = Δ*_i_*, $\Psi_{ik}=\left(1-\Delta_{i}\right)\psi_{k},k=1,\ldots ,K$, and $\Psi_{iK+1}=\left(1-\Delta_{i}\right)\left(1-\sum_{k=1}^{K}\psi_{k}\right)$.

Subjects can be partitioned into two groups with group 1 having $m_{i}^{*}>0$ and group 2 having $m_{i}^{*}=0$. The complete likelihood can then be expressed as:


$$\ell =\sum_{i\in \text{group}1}^{N}\sum_{k=1}^{K+1}{𝟙}_{c_{i}=k}\left(\ln(\Psi_{ik}\right)+\ell_{ik}^{1})+\sum_{i\in \text{group}2}^{N}\sum_{k=0}^{K+1}{𝟙}_{c_{i}=k}\left(\ln(\Psi_{ik}\right)+\ell_{ik}^{2}),$$

where $\ell_{ik}^{1}$ denotes $\ell_{ik}$ for group 1 and $\ell_{ik}^{2}$ denotes $\ell_{ik}$ for group 2. The complete likelihood is decomposed into three likelihood components.

In group 1, we have $m_{i}^{*}=m_{i}$. Thus, for any *k* = 1*,…,k* + 1,


$$\begin{array}{l} \ell_{ik}^{1}=\ln(f(y_{i},r_{i},m_{i}^{*}|x_{i},l_{i},c_{i}=k))\\ =\ln(f(y_{i},r_{i}|m_{i}^{*},x_{i},l_{i},c_{i}=k)f(m_{i}^{*}|x_{i},l_{i},c_{i}=k))\\ =\ln(f(y_{i}|m_{i}^{*},x_{i},l_{i},c_{i}=k)f(r_{i}|m_{i}^{*},x_{i},l_{i},c_{i}=k)f(m_{i}^{*}|x_{i},c_{i}=k))\\ =\ln(f(y_{i}|m_{i}^{*},x_{i}))+\ln(f(m_{i}^{*}|x_{i},c_{i}=k))\\ =-0.5\ln(2\pi )-\ln(\delta )-\frac{{\left(y_{i}-\beta_{0}-\beta_{1}m_{i}^{*}-\beta_{2}-\left(\beta_{3}+\beta_{4}\right)x_{i}-\beta_{5}x_{i}m_{i}^{*}\right)}^{2}}{2\delta^{2}}+\ln(\text{Beta}(m_{i}^{*}|\mu_{k},\phi )). \end{array}$$

In group 2 where $m_{i}^{*}=0$, we have:


$$\begin{array}{l} \ell_{i0}^{2}=\ln(f(y_{i},r_{i}=0,m_{i}^{*}=0|x_{i},l_{i},c_{i}=0))=\ln(f(y_{i},r_{i}=0|x_{i},l_{i},c_{i}=0))\\ =\ln(f(y_{i}|x_{i},l_{i},c_{i}=0)f(r_{i}=0|x_{i},l_{i},c_{i}=0))\\ =\ln(f(y_{i}|x_{i},l_{i},c_{i}=0))\\ =-0.5\ln\;(2\pi )-\ln\;(\delta )-\frac{{\left(y_{i}-\beta_{0}-\beta_{3}x_{i}\right)}^{2}}{2\delta^{2}}, \end{array}$$

And for any *k* = 1*,…,k* + 1,


$$\begin{array}{l} \ell_{ik}^{2}=\ln(f(y_{i},r_{i}=0,m_{i}^{*}=0|x_{i},l_{i},c_{i}=k))=\ln(f(y_{i},r_{i}=0|x_{i},l_{i},c_{i}=k))\\ =\ln(\int_{0}^{1/l_{i}}f(y_{i},r_{i}=0|x_{i},l_{i},c_{i}=k,m)dF(m|x_{i},l_{i},c_{i}=k))\\ =\ln(\int_{0}^{1/l_{i}}f(y_{i}|x_{i},m)dF(m|x_{i},l_{i},c_{i}=k))\\ =\ln(\int_{0}^{1/l_{i}}f(y_{i}|x_{i},m)f(m|x_{i},l_{i},c_{i}=k))dm\\ =-0.5\ln(2\pi )-\ln(\delta )+\ln(\int_{0}^{1/l_{i}}h(y_{i}|x_{i},m)\text{Beta}(m|\mu_{k},\phi ))dm, \end{array}$$

where


$$h(y_{i}|x_{i},m)=\exp\left(-\frac{{\left(y_{i}-\beta_{0}-\beta_{1}m-\beta_{2}-\left(\beta_{3}+\beta_{4}\right)x_{i}-\beta_{5}x_{i}m\right)}^{2}}{2\delta^{2}}\right).$$

### 2.3 EM algorithm

The Expectation-Maximization (EM) algorithm (Dempster et al., 1977) is used to obtain the maximum likelihood estimates of parameters. Throughout this iterative estimation procedure, we let Θ denote the full parameter vector containing all parameters and let Θ^0^ denote the initial parameter values. At each iteration, the conditional expectation of the complete-data log-likelihood is computed given the observed data.

#### 2.3.1 E-Step

The conditional expectation of the log-likelihood function is defined as follows:


$$Q(\text{Θ}|{\text{Θ}}^{0})=E[\ell |m_{i}^{*},y_{i},r_{i},l_{i},{\text{Θ}}^{0}]=\sum_{i\in \text{group}1}^{N}\sum_{k=1}^{K+1}E[{𝟙}_{c_{i}=k}|m_{i}^{*},y_{i},r_{i}=1,l_{i},{\text{Θ}}^{0}](\ln({\text{Ψ}}_{ik})+\ell_{ik}^{1})+\sum_{i\in \text{group}2}^{N}\sum_{k=0}^{K+1}E[{𝟙}_{c_{i}=k}|m_{i}^{*},y_{i},r_{i}=0,l_{i},{\text{Θ}}^{0}](\ln({\text{Ψ}}_{ik})+\ell_{ik}^{2}).$$

Let $\tau_{ik}^{1}\left({\text{Θ}}^{0}\right)=E[{𝟙}_{c_{i}=k}|m_{i}^{*},y_{i},r_{i}=1,l_{i},{\text{Θ}}^{0}]$ and $\tau_{ik}^{2}\left({\text{Θ}}^{0}\right)=E[{𝟙}_{c_{i}=k}|m_{i}^{*},y_{i},r_{i}=0,l_{i},{\text{Θ}}^{0}]$.


$$\begin{array}{l} \tau_{ik}^{1}\left({\text{Θ}}^{0}\right)=E[{𝟙}_{c_{i}=k}|m_{i}^{*},y_{i},r_{i}=1,l_{i},x_{i},{\text{Θ}}^{0}]=P(c_{i}=k|m_{i}^{*},y_{i},r_{i}=1,l_{i},x_{i},{\text{Θ}}^{0})\\ \;=\frac{f(m_{i}^{*},y_{i},r_{i}=1|\Theta^{0},\;c_{i}=k,l_{i},x_{i})\;P(c_{i}=k|\Theta^{0})}{\sum_{k=1}^{K+1}f(m_{i}^{*},y_{i},r_{i}=1|\Theta^{0},c_{i}=k,l_{i},x_{i})\;P(c_{i}=k|\Theta^{0})}\\ \;=\frac{{{\text{Ψ}}_{ik}\exp(\ell_{ik}^{1})|}_{\text{Θ}={\text{Θ}}^{0}}}{\sum_{k=1}^{K+1}{\text{Ψ}}_{ik}\exp(\ell_{ik}^{1}){|}_{\text{Θ}={\text{Θ}}^{0}}} \end{array}$$

$$\begin{array}{l} \tau_{ik}^{2}\left({\text{Θ}}^{0}\right)=E[{𝟙}_{c_{i}=k}|m_{i}^{*}=0,y_{i},r_{i}=0,l_{i},x_{i},{\text{Θ}}^{0}]=P(c_{i}=k|m_{i}^{*}=0,y_{i},r_{i}=0,l_{i},x_{i},{\text{Θ}}^{0})\\ \;=\frac{f(y_{i},r_{i}=0|\Theta^{0},\;c_{i}=k,l_{i},x_{i})\;P(c_{i}=k|\Theta^{0})}{\sum_{k=0}^{K+1}f(y_{i},r_{i}=0|\Theta^{0},c_{i}=k,l_{i},x_{i})\;P(c_{i}=k|\Theta^{0})}\\ \;=\frac{{{\text{Ψ}}_{ik}exp\;(\ell_{ik}^{2})|}_{\text{Θ}={\text{Θ}}^{0}}}{\sum_{k=0}^{K+1}{\text{Ψ}}_{ik}exp\;(\ell_{ik}^{2}){|}_{\text{Θ}={\text{Θ}}^{0}}} \end{array}$$

Thus, we have:


$$\begin{array}{l} Q(\text{Θ}|{\text{Θ}}^{0})=E[\ell |m_{i}^{*},y_{i},r_{i},l_{i},{\text{Θ}}^{0}]=\sum_{i\in \text{group}1}^{N}\sum_{k=1}^{k+1}\tau_{ik}^{1}\left({\text{Θ}}^{0}\right)\left(\ln\;({\text{Ψ}}_{ik}\right)+\ell_{ik}^{1})\\ \;+\sum_{i\in \text{group}2}^{N}\sum_{k=0}^{k+1}\tau_{ik}^{2}\left({\text{Θ}}^{0}\right)\left(\ln\;({\text{Ψ}}_{ik}\right)+\ell_{ik}^{2}). \end{array}$$

#### 2.3.2 M-Step

In the M-step, *Q*(Θ | Θ^0^) is maximized with respect to Θ to obtain updated parameter estimate denoted as Θ^1^. The E-step and M-step are repeated until the Euclidean distance between successive parameter estimates |Θ^0^ − Θ^1^|< 10^−8^. The final estimate Θ^1^ is taken as the maximum likelihood estimator (MLE).

#### 2.3.3 Information matrix

Let Θ^*^ denote the MLE. The information matrix is computed as below which can be used to obtain standard errors (Oakes, 1999).


$$I=-{\frac{\partial Q(\text{Θ}|{\text{Θ}}^{*})}{\partial {\text{Θ}}^{2}}|}_{\text{Θ}={\text{Θ}}^{*}}-{\frac{\partial Q(\text{Θ}|{\text{Θ}}^{0})}{\partial \text{Θ}\partial {\text{Θ}}^{0}}|}_{\text{Θ}={\text{Θ}}^{*},{\text{Θ}}^{0}={\text{Θ}}^{*}}$$

## 3 Results

### 3.1 Simulation

Simulation studies were conducted to assess the performance of the ZIBM model. Two settings were considered. In the first setting, data were generated from a univariate beta mixture distribution to evaluate the proposed model under correct model specification. The performance of the ZIBM model was compared with the nonparametric causal mediation approach of Imai, Keele, and Tingley (Imai et al., 2010) (hereafter referred to as the IKT approach). Although the IKT method adopts a similar definition of the natural indirect effect (NIE) compared to ours, it was not developed for microbiome data and was expected to perform poorly in the presence of zero inflation. In the second setting, the ZIBM was applied to simulated microbiome data with many taxa. The objective was to identify significant mediators, and the results were compared with those obtained from CCMM (Sohn and Li, 2019), which was specifically developed for microbiome data.

In both settings, the exposure *X* was generated from a Bernoulli distribution, Ber(0.5). False zeros were introduced based on the LOD mechanism in (Equation 5) of Subsection 2.1.1. The library sizes were randomly sampled from a real study (Arthur et al., 2013) (see Section 3.2 for more details), ranging from 31,607 to 911,652. In each setting, 100 datasets were generated to evaluate model performance. Asymptotic variances were used to compute coverage probabilities for each parameter, and the multivariate delta method was employed to derive standard errors for the mediation effects.

#### 3.1.1 Simulation setting I

In this setting, the mediator *M* was generated from a univariate beta mixture distribution using (Equations 2-4). The outcome *Y* was generated from (Equation 1). Two simulation scenarios were considered with different numbers of beta mixture components for the mediator. The first scenario had three beta mixture components with 300 samples in each of the 100 datasets. In this scenario, 41.3% of the observed *M* values were zero, among which 44.6% were false zeros generated from the LOD mechanism. The second scenario had two beta mixture components with 200 samples in each of the 100 datasets. In this scenario, 51.2% of the observed *M* values were zero, among which 75.6% were false zeros. The results were shown in **Table 1**. From the results, we can see that both scenarios had accurate estimates of the unknown parameters and mediation effects. The bias of all the parameters was small. Some parameters had higher bias percentage due to small true values. Note that the bias percentage of *α*_01_ in the three-component beta mixture model is not available since the true value is 0. The coverage probability (CP) from the 95% CIs was close to 95%. On the other hand, IKT approach had relatively poor performance with respect to both CP and bias compared to ZIBM. The bias was likely due to two factors: (i) failure to account for false 190 zeros and (ii) the lack of NIE decomposition into NIE_1_ and NIE_2_.

**Table 1:** Simulation results for mediators generated from three- and two-beta mixture distributions.

	Three-beta mixture distribution						Two-beta mixture distribution					
Parameter	True	Mean estimate	Bias	Bias (%)	Mean SE	CP (%)	True	Mean estimate	Bias	Bias (%)	Mean SE	CP(%)
ZIBM												
NIE_1_	-0.28	-0.27	0.01	-2.23	0.17	92	-0.18	-0.18	-0.00	0.75	0.16	97
NIE_2_	0.57	0.53	-0.04	-7.54	0.36	94	0.57	0.60	0.03	5.65	0.46	96
NIE	0.29	0.25	-0.04	-12.66	0.45	95	0.39	0.42	0.03	7.87	0.58	95
*β*_0_	-5.00	-5.01	-0.01	0.22	0.20	92	-5.00	-4.97	0.03	-0.55	0.23	95
*β*_1_	10.00	9.94	-0.06	-0.56	0.45	89	10.00	9.96	-0.04	-0.39	0.32	99
*β*_2_	8.00	8.03	0.03	0.39	0.23	94	8.00	7.97	-0.03	-0.36	0.27	94
*β*_3_	1.00	1.16	0.16	15.68	0.31	92	1.00	0.93	-0.07	-6.92	0.38	91
*β*_4_	1.00	0.80	-0.20	-20.09	0.35	95	1.00	1.09	0.09	8.60	0.42	90
*β*_5_	1.00	1.08	0.08	8.48	0.71	94	1.00	1.02	0.02	1.94	0.49	94
*γ*_0_	-1.50	-1.52	-0.02	1.11	0.21	98	-1.50	-1.50	0.00	-0.14	0.26	98
*γ*_1_	-0.50	-0.45	0.05	-9.02	0.33	93	-0.50	-0.54	-0.04	7.62	0.41	96
*ϕ*	10.00	11.05	1.05	10.49	2.48	94	10.00	10.66	0.66	6.55	1.88	94
*δ*	1.00	1.02	0.02	2.17	0.04	92	1.00	0.99	-0.01	-1.18	0.05	94
*α*_01_	0.00	0.00	0.00	–	0.16	93	1.00	1.02	0.02	2.28	0.14	94
*α*_02_	-2.00	-2.01	-0.01	0.69	0.26	96	-5.00	-5.04	-0.04	0.72	0.25	95
*α*_03_	-5.00	-5.11	-0.11	2.10	0.32	96	–	–	–	–	–	–
*α*_11_	-0.50	-0.48	0.02	-3.60	0.22	92	-0.50	-0.52	-0.02	4.62	0.18	93
*α*_12_	-0.50	-0.49	0.01	-2.75	0.37	95	-0.50	-0.51	-0.01	1.94	0.31	95
*α*_13_	-0.50	-0.45	0.05	-10.72	0.51	92	–	–	–	–	–	–
*ψ*_1_	0.20	0.20	0.00	1.73	0.03	96	0.30	0.30	0.00	0.03	0.04	95
*ψ*_2_	0.30	0.30	-0.00	-0.69	0.05	91	–	–	–	–	–	–
IKT												
NIE	0.29	-0.26	-0.55	-190.35	0.23	29	0.39	-0.14	-0.53	-136.54	0.47	79

The mean of estimates, bias, percentage of bias, mean of asymptotic standard error, and coverage probability from 95% CIs for mediation effects and each parameter are reported. The mediation effect from the IKT approach was also reported at the bottom.

#### 3.1.2 Simulation setting II

In this simulation setting, we proposed an approach to generate microbiome RA data from a Dirichlet distribution, with only the first two taxa correlated with the covariate *X*. Therefore, based on the definition of mediation effect in Subsection 2.1.2, only the first two taxa have non-zero NIEs. The steps to generate RA for each taxon with *K* + 1 taxa are provided in Supplementary Material.

The outcome *Y* model was generated as follows:


$$Y=\beta_{0}+\beta_{1}M_{1}+\beta_{2}{𝟙}_{M_{1}>0}+\beta_{3}X+\beta_{4}X{𝟙}_{M_{1}>0}+\beta_{5}XM_{1}+\epsilon$$

where *M*_1_ denotes the first taxon. The outcome *Y* was marginally associated with the first taxon, and due to the compositional structure, outcome *Y* was also marginally associated with other taxa. (*β*_0_*, β*_1_*, β*_2_*, β*_3_*, β*_4_*, β*_5_) = (4, 100, 2, 1, 1, 1) and *ϵ* follows a standard normal distribution. We set *γ*_0_ = 0 and *γ*_1_ = −3 for the first taxon, and for other taxon *γ*_0_ was adjusted to make their zero percentage close to 50%. *γ*_1_ for the other taxon was set to 0 to make sure their NIE_2_ was 0. The *α* was adjusted to ensure that approximately 40% of all zeros in the first taxon were false zeros.

Model performance was evaluated using three metrics: Recall, Precision, and F1, defined as follows:


$$\text{Recall}=\frac{TP}{TP+FN},\text{Precision}=\frac{TP}{TP+FP},\text{F}1=\frac{2}{\frac{1}{\text{Recall}}+\frac{1}{\text{precision}}}$$

where TP, TN, FP, FN are true positive, true positive, false positive, and false negative, respectively. Multiple testing in our model was adjusted using the Benjamini-Hochberg procedure (Benjamini and Hochberg, 1995) with false discovery rate (FDR) set to 20%. Accordingly, the target precision rate was 80%.

The results for simulation setting II are presented in **Table 2**. CCMM, which was specifically developed for microbiome datasets, was included for comparison. The proposed method achieved substantially better performance, reaching the target precision rate with a reasonable recall, whereas CCMM exhibited lower recall and remarkably lower precision. In the 300 taxon case, CCMM failed to produce results due to computational challenge.

**Table 2:** Simulation results for the simulation setting II with comparison to CCMM.

*K* + 1	n	Recall (%)			Precision (%)			F1 (%)		
		ZIBM (NIE_1_)	ZIBM (NIE_2_)	CCMM	ZIBM (NIE_1_)	ZIBM(NIE_2_)	CCMM	ZIBM (NIE_1_)	ZIBM (NIE_2_)	CCMM
10	200	80.00	84.80	70.50	97.70	83.50	19.60	84.90	77.10	30.50
10	300	82.50	86.00	72.00	93.30	87.50	17.60	84.10	79.00	28.20
25	200	87.50	85.00	69.00	80.40	90.80	16.40	80.20	80.10	26.20
25	300	98.00	86.00	68.50	91.40	92.80	14.10	93.30	80.70	23.30
50	200	97.50	91.90	67.50	99.30	95.30	14.60	97.90	88.60	23.70
50	300	88.80	98.00	68.00	96.60	87.80	12.40	90.50	90.30	20.80
100	200	83.50	95.00	79.00	89.90	91.70	17.30	83.20	89.30	27.70
100	300	90.90	96.00	83.00	85.80	90.40	14.20	84.90	90.10	24.00
300	200	97.00	96.00	–	94.70	83.80	–	94.70	85.10	–
300	300	99.50	96.80	–	99.30	91.10	–	99.20	89.70	–

Under Simulation Setting II, we additionally compared ZIBM with a no-mixture model, which follows the MarZIC specification (Wu et al., 2022) by using a univariate beta distribution for the nonzero component in (Equation 2) instead of a beta mixture distribution. We also included CCMM in the comparison. To highlight the difference between the mixture and no-mixture models, we set *γ*_1_ = 0, so that Δ was independent of *X*, and therefore NIE_1_ was mainly driven by *µ*, according to Subsection 2.1.2. These results are presented in **Table 3**. In this setting, we set the RA of the first two taxa to be around 0.1 to amplify the effect of the mixture component. Overall, MarZIC showed lower recall than ZIBM, suggesting that the single-beta specification may lose power when the nonzero abundance distribution has a mixture structure. However, its precision remained similar to that of ZIBM. CCMM showed lower recall than ZIBM and lower precision than both ZIBM and MarZIC.

**Table 3:** Simulation results for the simulation setting II with comparison to no-mixture model (MarZIC) and CCMM.

	Recall (%)			Precision (%)			F1 (%)			Type 1 (%)	
*K* + 1	MarZIC	ZIBM	CCMM	MarZIC	ZIBM	CCMM	MarZIC	ZIBM	CCMM	MarZIC	ZIBM
10	58.50	97.50	58.50	95.50	95.33	88.80	70.40	95.63	66.80	0.40	0.60
25	53.50	92.00	53.50	90.90	91.19	79.50	65.57	89.25	59.60	0.12	0.08
50	53.00	93.00	54.00	82.03	82.10	64.70	60.99	84.12	53.70	0.04	0.06
100	50.50	88.50	53.50	92.83	93.17	42.60	64.57	88.23	44.30	0.49	0.62
300	50.00	77.50	–	99.33	98.67	–	66.40	84.33	–	6.61	6.70

### 3.2 Real data application

In this section, we applied our proposed method to a study conducted by Arthur et al (Arthur et al., 2013) to investigate the effect of VSL#3 on inflammation-associated CRC in mice after the induction of inflammation. VSL#3 is a probiotic medical supplement that was formulated to alter the microflora of the gut and help manage severe digestive conditions. It is a cocktail (Sigma-Tau Pharmaceuticals, Inc.) of eight strains of lactic acid-producing bacteria: *Lactobacillus plantarum, Lactobacillus delbrueckii subsp. Bulgaricus, Lactobacillus paracasei, Lactobacillus acidophilus, Bifidobacterium breve, Bifidobacterium longum, Bifidobacterium infantis*, and *Streptococcus salivarius subsp*. Recent studies have shown that orally administered VSL#3 reduced intestinal inflammation in humans (Gionchetti et al., 2000; Sood et al., 2009; Madsen et al., 2001; Pagnini et al., 2010). The underlying mechanisms are not well understood, but are hypothesized to both directly reduce inflammation through probiotic activity and indirectly by altering gut microbiota.

The 24-week study used 24 mice, with 10 in the oral treatment group and 14 in the control group. The treatment group received a standard baseline dose of orally administered VSL#3 probiotic starting on the first day of receiving injections of azoxymethane to induce inflammation-associated CRC. For six weeks, the mice in the treatment group received 109 colony-forming units of probiotic every weekday, which is a commonly administered dose in veterinary medicine (Nataraj et al., 2024; Li et al., 2026). The microbiome data were obtained from 16S rRNA sequencing with samples collected from stool at the end of the study. For each operational taxonomic unit (OTU) obtained from the sample, RA was analyzed as a mediator using our model. The outcome variable was dysplasia score, an ordinal categorical variable measuring the abnormality of cell growth, with higher scores indicating greater dysplasia. The highest score indicated evidence of colon cancer. Two blinded experts reviewed the histology of colon tissue for inflammation and dysplasia or precancerous lesions.

Arthur et al. (2013), Wu et al. (2022), and our study investigated the compositional microbiome mediators in the association between probiotic use and dysplasia score. Arthur et al. (2013) found that depletion of the *Clostridium* bacterial group was associated with VSL#3 administration and tumorigenesis using mediation by linear models analysis. Applying MarZIC, Wu et al. (2022) identified two significant mediators (i.e., family *S24–7* and class *Bacilli*). In the same VSL#3 analysis, CCMM (Sohn and Li, 2019) and IKT (Imai et al., 2010) methods did not identify significant mediation effects for the OTUs (Wu et al., 2022). In contrast, the ZIBM model proposed in this study identified 10 significant mediators, where two belonged to the *Bacilli* class, one from the *Bacteroidaceae* family, one from the *Lachnospiraceae* family, one from *Verrucomicrobiaceae* family, and five from the *S24–7* family. See **Figure 2** for the heatmap of these results.

**Figure 2:**
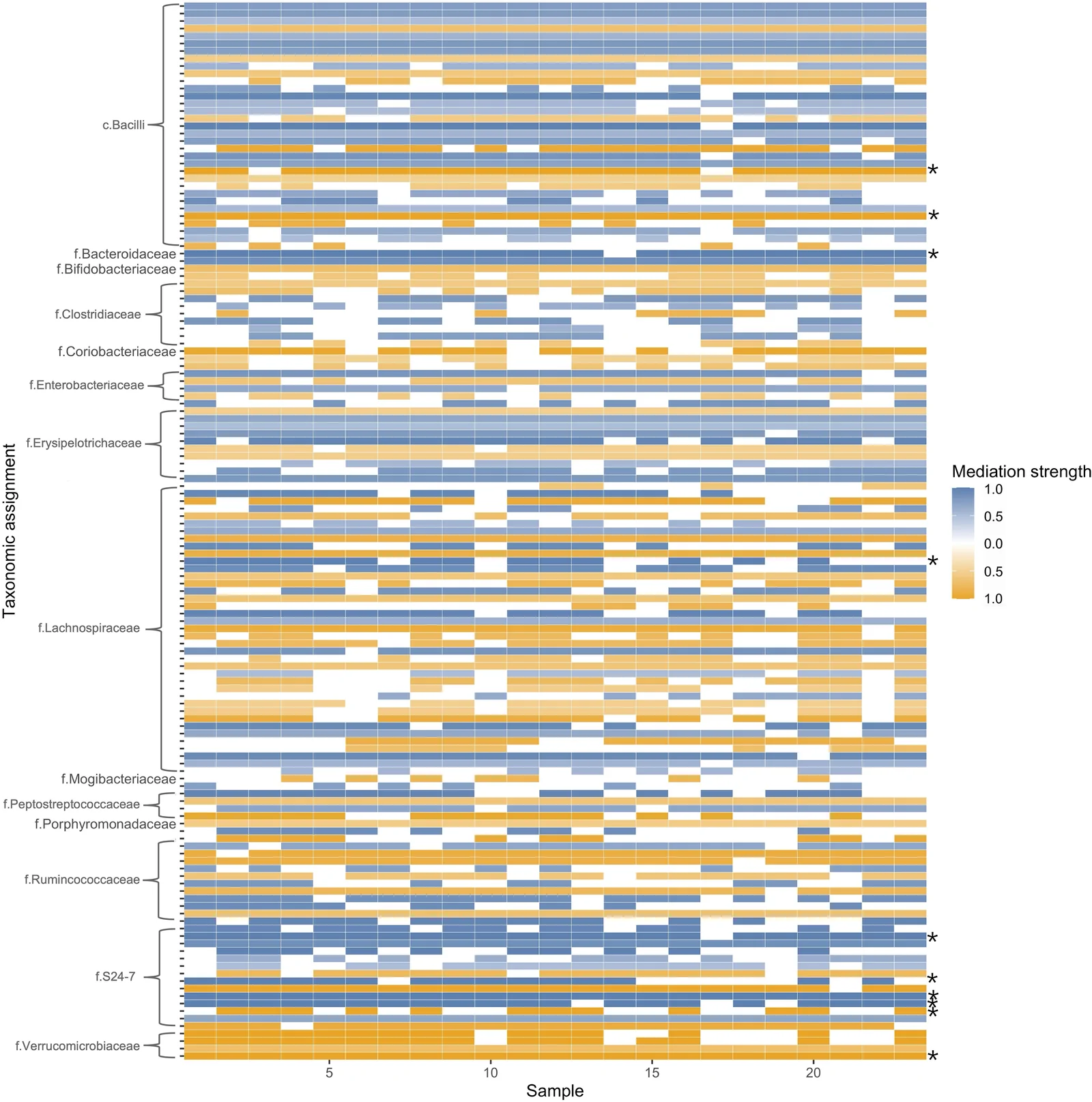
Heatmap of mediation effects based on NIE_1_ in the VSL#3 study. Yellow indicates a negative (NIE_1_), and blue indicates a positive (NIE_1_). An asterisk indicates a significant mediator. Taxonomic assignments are labeled on the vertical axis. Some rows share the same taxon name because they correspond to different OTUs assigned to the same taxon. Samples are labeled on the horizontal axis. Absence of an OTU in a sample is shown as blank in the heatmap.

The literature supports these findings. Notable *Bacilli*-class members implicated in CRC (Gagnière et al., 2016; Karpiński et al., 2022). *Bacteroidaceae* has been found to be significantly enriched in stool samples from patients with inflammatory bowel disease and CRC (Dan et al., 2025). *Lachnospiraceae* has a complex and relatively well-studied association with gut inflammatory disease and CRC. The literature suggests that specific genera of bacteria within the family are protective commensal intestinal symbionts while others are pathogenic (Zhang et al., 2023; Ma et al., 2024; Shah et al., 2025; Tang et al., 2026). *Verrucomicrobiaceae* is not as well-characterized. However, one of its strains has been shown to have a context-dependent role in CRC pathogenesis. In inflammatory disease contexts, one of its members, *Akkermansia muciniphila*, acts as a pro-tumorigenic bacteria whereas it is enriched in sporadic CRC tissue and one of the main differentiating phyla in cancer patients compared to healthy controls (Soheilipour et al., 2025). Another recent murine study, however, identified *Akkermansia muciniphila* as a potentially protective bacteria due to its role in reducing chronic stress (Jin et al., 2025). The *S24–7* family has been associated with murine colitis, however, the clinical relevance in humans is yet unknown (Lagkouvardos et al., 2019).

## 4 Discussion

In this paper, we propose a ZIBM model for mediation analysis with compositional microbiome mediators. The model extends the MarZIC framework by introducing a mixture representation for the non-zero component, which accounts for excess zeros and latent heterogeneity in microbiome RA data. It is formulated within the potential-outcomes framework and provides interpretable estimates of microbiome-mediated causal effects.

Simulation results show that the proposed method performs well under conditions commonly observed in microbiome data, including zero inflation, compositionality, and latent heterogeneity. Compared with existing methods (i.e., IKT (Imai et al., 2010) and CCMM (Sohn and Li, 2019)), the ZIBM model yields more reliable estimates when false zeros are present or when standard distributional assumptions are violated. The real data analysis demonstrates the utility of the proposed model for microbiome studies with sparse and compositional mediators. The identified mediators are associated with disease-related outcomes while accounting for sparsity and compositional constraints. Compared to the MarZIC method’s application of the Arthur et al. (2013) study, the ZIBM framework identified eight additional bacterial families as causal mediators (Wu et al., 2022). All of the bacterial classes and families identified have been associated with high-risk inflammatory diseases and CRC. However, the associations are complex with different genera within one family having different commensal or pathogenic effects. Therefore, further research with specific targeted genera and species using the ZIBM framework is warranted. Our findings suggest that ZIBM may be a more sensitive approach that allows for greater capture of potential causal mediators. Identifying causal mediators that explain how or why an exposure affects an outcome will help physicians, researchers, and policymakers better understand oncogenesis, discover risk factors, and develop interventions. In this clinical scenario, identifying the specific taxa of bacteria that are differentially associated with probiotic use and carcinogenesis can allow for risk stratification, early detection, and personalized treatment.

The proposed model offers several advantages. The zero-inflated beta mixture formulation accounts for both structural and sampling zeros and captures heterogeneity in non-zero RAs. It provides an interpretable mediation framework under the potential-outcomes paradigm for sparse and compositional microbiome data. The ZIBM framework also has several limitations. The current ZIBM model estimates the marginal mediation effect of each taxon separately. Therefore, it does not directly test interactions between taxa. It also cannot determine whether a significant taxon is the primary mediator or is associated with another causal taxon. Measured confounders can be included as covariates in the model, but taxon-taxon interactions and unmeasured confounding are not addressed in the current framework. Thus, significant taxa identified by ZIBM should be interpreted as candidate marginal mediators. The number of mixture components needs to be specified in advance, which may affect model performance, and computational burden may increase in high-dimensional microbiome settings. Future work includes data-driven selection of mixture components. In summary, the ZIBM model provides a practical approach for mediation analysis with zero-inflated compositional microbiome mediators.

## Data Availability

The data analyzed in this study is subject to the following licenses/restrictions: The original dataset was obtained from a previously published study, which is cited in the manuscript. Access to the original data is subject to the terms and restrictions of the original data source. The pre-processed dataset analyzed in this study is available from the corresponding authors upon reasonable request, where permitted by the original data use terms. Requests to access these datasets should be directed to seungjun.ahn@mountsinai.org and zhigang.li@ufl.edu. The dataset analyzed in this paper is available upon request. R package is freely available on GitHub repository at https://github.com/fuweijia/ZIBM.
