# Supplementary material for: A zero-inflated beta mixture model for marginal mediation analysis with compositional microbiome mediators
Source: Front Cell Infect Microbiol. 2026 Oct 29;16:1877170. doi: 10.3389/fcimb.2026.1877170 (PMC13754734; doi:10.3389/fcimb.2026.1877170)
Supplement: Supplementary file 1 [file DataSheet1.pdf]

Supplementary Material

1 DATA GENERATION PROCESS

Simulated data are adapted from the MarZIC (Wu et al., 2022) framework, with modifications described below. Let $Q+1$ be the number of taxa, for the ith subject, the microbiome data generation steps are listed below:

Step 1: Decide which component of the kth component mixture will be used for this subject. For illustration purpose, k is set to be 2. In the simulation, the weights of the 2-component mixture are $\psi_1 = 0.3$ and $\psi_2 = 0.7$. Generate a Bernoulli random variable from $\mathrm{Ber}(0.3)$. If $\mathrm{Ber}(0.3) = 1$, the first component is used. Otherwise, the second component is used.

Step 2: Generate true zeros for all taxa. The zeros for a taxon were generated using a $\mathrm{Ber}(\Delta)$. So the probability of the taxon being 0 is equal to Δ. For taxon 1, we set $\Delta = 0$ so that there is no zero in taxon 1. For taxon 2, Δ was calculated using the selected mixture component from Step 1. For all other taxa, γ_0 were generated $\mathrm{Unif}(1, 2)$ and $\gamma_1 = 0$ for Δ. Thus, only the absence (or presence) of taxon associated with X. The total percentage of zeros was between 68.8$\%$ and 81.6$\%$ with $K+1$ ranging from 10 to 500, which indicates high data sparsity.

Step 3: Generate RA for the non-zero taxa from a Dirichlet distribution. Assume we had P non-zero taxa (from Step 1) indexted by $(t_1, t_2, \ldots, t_p)$ in the ascending order meaning $t_1 < \cdots < t_p$. Here $t_1 = 1$ since the first taxon does not have any zeros from Step 1. The RA of those non-zero taxa was generated by the P-dimensional Dirichlet distribution with the dispersion parameter ϕ and mean parameter $(\mu_{t_1}, \cdots, \mu_{t_p})$ that satisfies $\sum_{p=1}^{p} \mu_{t_p} = 1$. The dispersion parameter ϕ was set to be 10 to mimic the overdispersion in real data. The values of mean parameters were chosen in a way such that it has smaller values for taxa with larger Δ's in Step 1 so that taxa with lower abundance are more likely to have zeros. More specifically, the mean parameters were determined as follows: If $t_2 = 2$ (i.e., taxa 2 is one of the non-zero taxa):

$$\mu_{t_1} = \frac{\exp(\alpha_0^1)}{\sum_{q=1}^{Q+1}\exp(\alpha_0^q)} \times \frac{1}{1+\exp(\alpha_0 + \alpha_1 X)},$$

$$\mu_{t_2} = \frac{\exp(\alpha_0^1)}{\sum_{q=1}^{Q+1}\exp(\alpha_0^q)} \times \frac{\exp(\alpha_0 + \alpha_1 X)}{1+\exp(\alpha_0 + \alpha_1 X)},$$

$$\mu_{t_p} = \left(1 - \frac{\exp(\alpha_0^1)}{\sum_{q=1}^{Q+1}\exp(\alpha_0^q)}\right) \times \frac{\exp(\alpha_0^{t_p})}{\sum_{p=3}^{P}\exp(\alpha_0^{t_p})}, p \in \{3, \ldots, P\},$$

where $\alpha_0 = -2$, $\alpha_1 = 5$, α_0^1 was set to be the value such that the false zeros for taxon 2 that will be generated in next step will be around 20%, and α_0^k, $k \in \{2, \ldots, K+1\}$ were generared from $\mathrm{Unif}(0, 1)$.

If $t_2 > 2$ (i.e., taxa 2 is not one of the non-zero taxa):

$$\mu_{t_1} = \frac{\exp(\alpha_0^1)}{\sum_{q=1}^{Q+1} \exp(\alpha_0^q)},$$
$$\mu_{t_p} = \left(1 - \frac{\exp(\alpha_0^1)}{\sum_{q=1}^{Q+1} \exp(\alpha_0^q)}\right) \times \frac{\exp(\alpha_0^{t_p})}{\sum_{p=2}^{P} \exp(\alpha_0^{t_p})}, p \in \{2, \ldots, P\},$$

Under this data generation, only the RA of taxa 1 and taxa 2 were dependent on X. The RA of taxa 3 to taxa K were independent of X.

Step 4: Generate sample absolute abundance (SAA) and false zeros from a multinomial distribution. Let $(\mathcal{R}_{t_1}, \ldots, \mathcal{R}_{t_p}$ denote the RA generated in Step 2 for the P non-zero taxa thus $\sum_{p=1}^{P} \mathcal{R}_{t_p} = 1$. The P-dimensional multinomial distribution with the parameter vector $(\mathcal{R}_{t_1}, \ldots, \mathcal{R}_{t_p}$ and the library size (randomly selected from the real data) was used to generate SAA for all the P non-zero taxa. Those taxa with SAA=0 generated from the multinomial distribution are false zeros.

Step 5: Getting final RA for all non-zero taxa. After SAA were generated for all non-zero taxa in Step 3, the SAA were divided by the library size to get the final RA for all non-zero taxa.

Step 6: Repeat the above Steps 1-5 for each subject to get a full data set of microbiome data for 200 subject.

REFERENCES

Wu, Q., O'Malley, J., Datta, S., Gharaibeh, R., Jobin, C., Karagas, M., et al. (2022). Marzic: A marginal mediation model for zero-inflated compositional mediators with applications to microbiome data. *Genes (Basel)* 13, 1049
